# Management Strategies for POSEIDON's Group 1

**DOI:** 10.3389/fendo.2019.00679

**Published:** 2019-10-01

**Authors:** Nikolaos P. Polyzos, Panagiotis Drakopoulos

**Affiliations:** ^1^Department of Reproductive Medicine, Dexeus University Hospital, Barcelona, Spain; ^2^Department of Surgical and Clinical Science, Faculty of Medicine and Pharmacy, Vrije Universiteit Brussel, Brussels, Belgium; ^3^Department of Clinical Medicine, Faculty of Health, Aarhus University, Aarhus, Denmark; ^4^Centre for Reproductive Medicine, Universitair Ziekenhuis Brussel, Vrije Universiteit Brussel, Brussels, Belgium

**Keywords:** poor responders, POSEIDON criteria, suboptimal responders, FSH receptor, LH, FSH polymorphisms, IVF

Poor ovarian responders represent one of the most difficult group of patients in every day clinical fertility practice. Still, a major limitation of the available published research is the striking diversity in the definitions used to define poor ovarian response, which could hamper the validity of the results (1, 2).

Despite the recent attempt by the European Society of Human Reproduction and Embryology (ESHRE) to apply a uniform definition for women who respond poorly to ovarian stimulation, the so called “Bologna” criteria (3), it seems that clinicians are still reluctant to use them in clinical studies (4), mainly due to the inability of these criteria to distinguish alterations in oocyte quantity vs. oocyte quality, grouping together women with different biologic characteristics and therefore altered clinical prognosis.

Recently, the POSEIDON group proposed a more detailed stratification of low responders, taking into account essential baseline characteristics of infertile women, which could have a significant impact on their reproductive outcome (5). In this context, patient classification is not only based on the number of oocytes retrieved, but also on various other features that may affect treatment success and should be carefully taken into consideration in the era of tailored-approach treatment, such as age and ovarian “sensitivity” to exogenous gonadotropins.

In this regard, four different patients' categories have been identified through the POSEIDON criteria, taking into account patients' age, ovarian reserve markers and response to stimulation in order to define patients' actual prognosis.

POSEIDON Group 1 apparently includes the best prognosis patients, compared to other POSEIDON groups, referring to young infertile women (<35 years old), with adequate ovarian reserve markers (AFC ≥ 5; AMH ≥ 1.2 ng/ml), and unexpected poor (<3 oocytes retrieved) or suboptimal (4–9 oocytes retrieved) response following conventional ovarian stimulation (5).

Management of women belonging to the POSEIDON group 1 requires a distinct diagnostic and therapeutic approach in relation to patients' characteristics, which should be specifically tailored to their young age and the adequate ovarian reserve of these women (6).

Age is undeniably the strongest determinant of treatment success in women seeking fertility advice (7). The age-related decline in fertility, owing to a significant decrease in both oocyte quantity (as reflected by lower oocyte yield) and quality (as reflected by higher aneuploidy and spontaneous abortion rates), is directly associated with the very low LBR observed in older women (8). Therefore, although prognosis is very bad in old poor responders, irrespective of the treatment modality used (9, 10), substantial benefit could be anticipated in younger women if an adequate number of oocytes is harvested. If we further consider that suboptimal response to stimulation significantly impairs cumulative live birth rates (11–13) and that women with unexpected poor/suboptimal responders may have better prognosis compared to patients with predicted low response (14–16), it could be stated that POSEIDON group 1 patients may represent the most interesting group, on which clinical research should focus in the future.

Several pathophysiological explanations have been proposed in order to clarify the nature of unexpected poor/suboptimal response. Ovarian sensitivity in relation to gonadotropin treatment has been the dominating theory, with evidence deriving from the investigation of the genetic variations of gonadotropins and their receptors (17). In particular, FSHR polymorphisms (e.g., Ser680Asn and Thr307Ala) have been associated with reduced sensitivity to gonadotropins (18) and may be the most reasonable explanation for the inadequate response following ovarian stimulation (19). This, in addition to the established need for higher gonadotropins in these patients (18), despite their normal ovarian reserve markers (20), suggests that genetic variation in the FSHR is a marker of ovarian sensitivity, irrespective of ovarian reserve.

On the other hand, a common variant of the β subunit of luteinizing hormone (LH) (v-LH) has been shown to affect FSH sensitivity and the ovarian response to FSH in normogonadotrophic women. Previous studies demonstrated that patients with this genetic variant of LH may experience an unexpected suboptimal response to stimulation and actually require higher cumulative-dose of gonadotropins (21, 22); thus, it may be imperative to consider the potential presence of such a genetic variant among several patients belonging to POSEIDON group 1.

Furthermore, less studied polymorphisms including FSH/LHCGR genes and their combinations may also be relevant, although evidence is sparse (23, 24).

Clear treatment guidelines have not been established for POSEIDON group 1 patients; still, this needs to be tailored in accordance to the underlying pathophysiological mechanism responsible for the impaired response to stimulation (6).

Utilization of higher gonadotropin doses of more “potent” recombinant formulations may be the solution in a significant percentage of these women, especially in the ones with polymorphisms identified in the FSHR gene. Taking into account that the Ser680Asn polymorphism of the FSHR gene may negatively influence the ovarian response to FSH stimulation and women with the genotype Ser/Ser appear to be more resistant to FSH action, a pharmacogenetic study demonstrated that treatment with higher FSH starting dose (225IU) in women homozygous for Ser680 (SS) resulted in similar serum estradiol (E2) levels with women who are homozygous for Asn680 (AA)/heterozygous (AS) treated with lower FSH doses (150IU) and significantly higher E2 levels compared to SS women treated with low 150IU dose (25). Moreover, a recent retrospective study evaluated the effect of FSH dose adjustment in women with a history of suboptimal response (4–9 oocytes retrieved) and demonstrated that an increase in the starting dose of FSH was significantly associated with a higher oocyte yield in the following IVF cycle (26).

On the other hand, administration of r-LH supplementation could be another option in these women, especially in cases of genetic variations of LH gene. Given the accumulating evidence from clinical research demonstrating that recombinant LH (rLH) could potentially increase the number of oocytes retrieved and result in higher pregnancy rates in women with non-pathological ovarian reserve tests and previous unexpected poor (27) or inadequate response (28), the use of rLH in these women is fully justified, and future research is essential to confirm these initial findings.

The utilization of novel promising approaches such a dual stimulation should not be overlooked and may be of benefit for POSEIDON group 1 patients. The rationale of this strategy is that poor prognosis women may undergo both follicular and luteal phase ovarian stimulation in the same menstrual cycle, in an attempt to maximize the number of oocytes retrieved and in turn increase the chance to obtain a genetically normal embryo in a short time interval (29). However, more evidence is needed for the applicability of luteal phase stimulation in poor responders, before implementation in clinical practice.

The synchronization of the follicular cohort through luteal phase estradiol/oral contraceptive pills (OCP) pre-treatment could be an option in young patients with unexpected poor or suboptimal response; albeit evidence extrapolated from studies in poor responders is controversial (2, 30).

Finally, adjuvant treatments with growth hormone (GH) or testosterone have been of great interest as an option to improve the outcome in women with a poor ovarian response and certainly merit evaluation in POSEIDON group 1. However, it should be stated that even if previous meta-analyses support the use of these regimens in poor responders (31, 32), results need to be interpreted with great caution due to limited evidence and small sample size of the relevant RCTs (33).

In conclusion, young women with normal ovarian reserve markers with a previous unexpected poor or suboptimal response seem to form a distinct group of infertile patients with different clinical prognosis compared to poor responders according to the “Bologna” criteria. Genetic polymorphisms of gonadotropins and their receptors may be a plausible explanation for the poor/suboptimal response following conventional ovarian stimulation; albeit more evidence is needed (NCT03007043, available at: clinicaltrials.gov). The management of these patients may imply the increase in the starting dose of recombinant FSH and/or supplementation with rLH or even double ovarian stimulation in an attempt to increase the number of oocytes retrieved and therefore the final reproductive outcome. The use of GH/testosterone and priming protocols including estradiol/OCPs represent other promising options. Nonetheless, further studies are warranted in order to validate these therapeutic approaches.

## Author Contributions

NP and PD contributed equally to the design and the writing of the manuscript.

### Conflict of Interest

The authors declare that the research was conducted in the absence of any commercial or financial relationships that could be construed as a potential conflict of interest.
